# Supplementary material for: Molecular basis of the lipid-induced MucA-MucB dissociation in Pseudomonas aeruginosa
Source: Commun Biol. 2020 Aug 3;3:418. doi: 10.1038/s42003-020-01147-1 (PMC7400510; doi:10.1038/s42003-020-01147-1)
Supplement: Supplementary file 1 — Supplementary information [file 42003_2020_1147_MOESM1_ESM.pdf]

Supplementary Information

Supplementary Figures

Supplementary Figure 1.

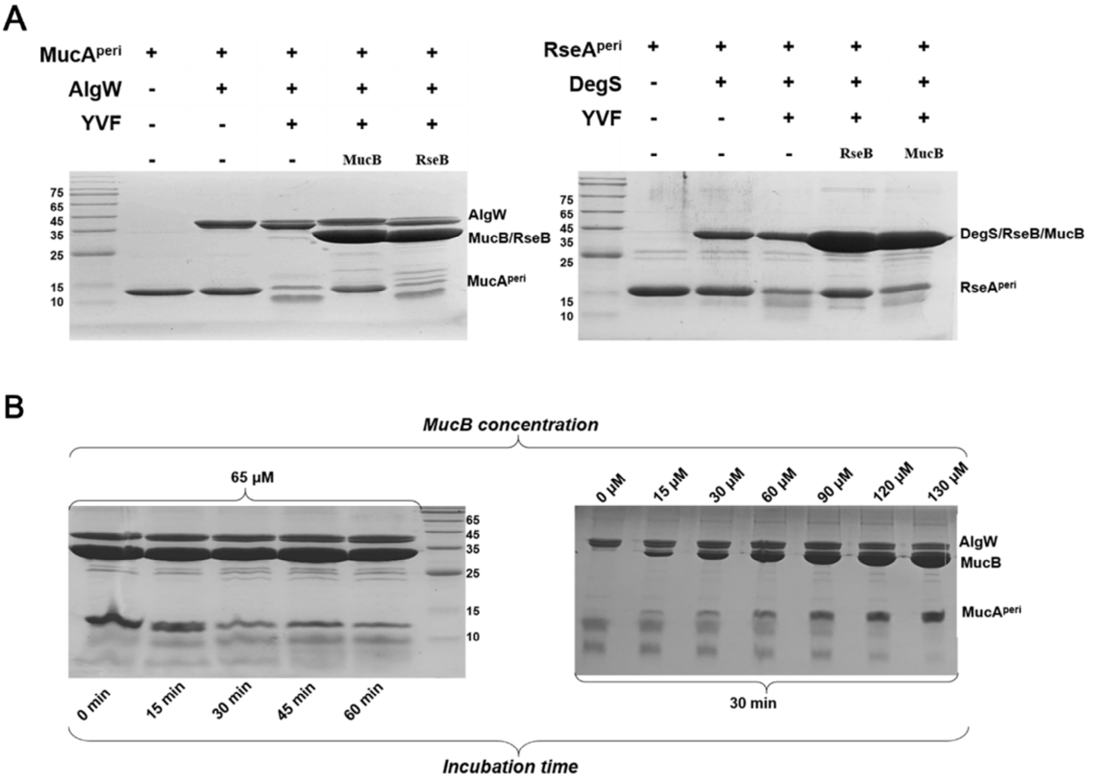

Supplementary Figure 1. MucB/RseB protects MucA^{peri}/RseA^{peri} from cleavage in a specific recognition pattern. (A) Left, SDS-PAGE analyses of MucA^{peri} (125 μM) degradation after the incubation of AlgW (25 μM), MucB (130 μM) and YVF peptide (80 μM). Right, RseA^{peri} (125 μM) was cleavaged by DegS (25 μM) in the presences of RseB (130 μM) and YVF peptide (80 μM). (B) The profile of the activated AlgW-mediated MucA^{peri} degradation in presence of different time incubation (left) or varying MucB concentration (right). In the degradation system, the concentrations of MucA^{peri} and AlgW are 125 μM and 25 μM, respectively.

Supplementary Figure 2.

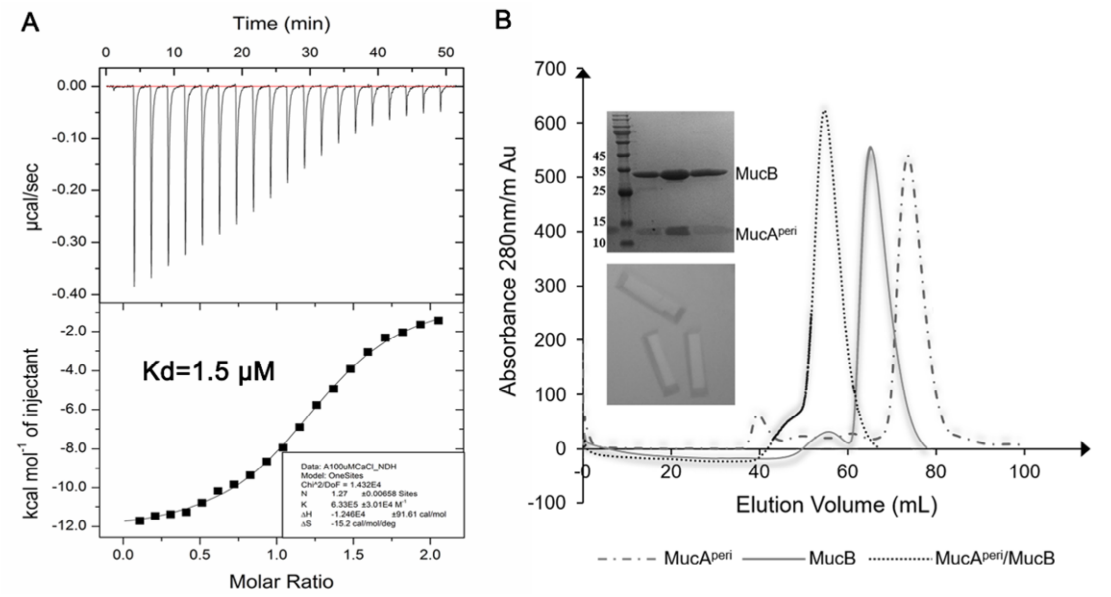

Supplementary Figure 2. (A) Isothermal titration calorimetry (ITC) analysis of MucA^{peri} and MucB interaction. (B) The overlayed size exclusion chromatography profiles (Superdex^{TM}-200, GE Healthcare) of MucA^{peri}, MucB and MucA^{peri}-MucB complex.

Supplementary Figure 3.

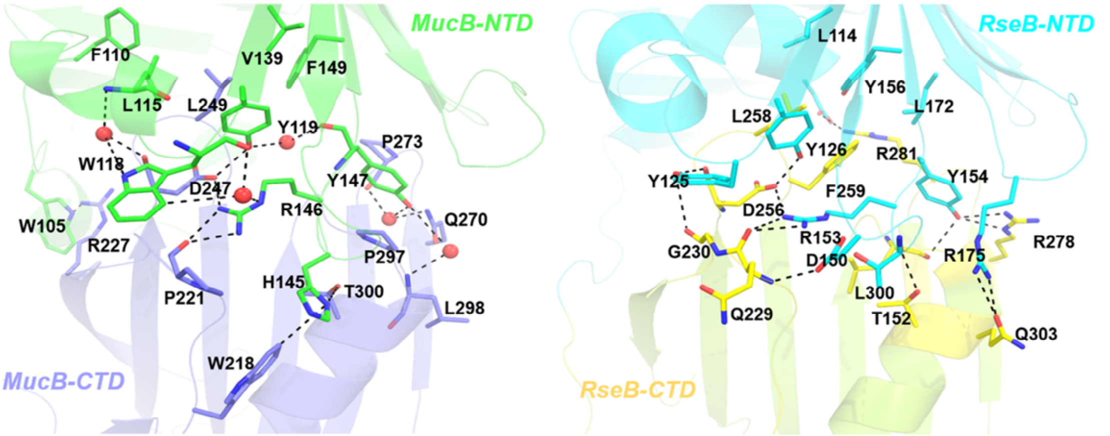

Supplementary Figure 3. The inter-domain interactions in MucB (Left) and RseB (Right). The key residues are shown with sticks, the water is shown with spheres and colored in red.

Supplementary Figure 4.

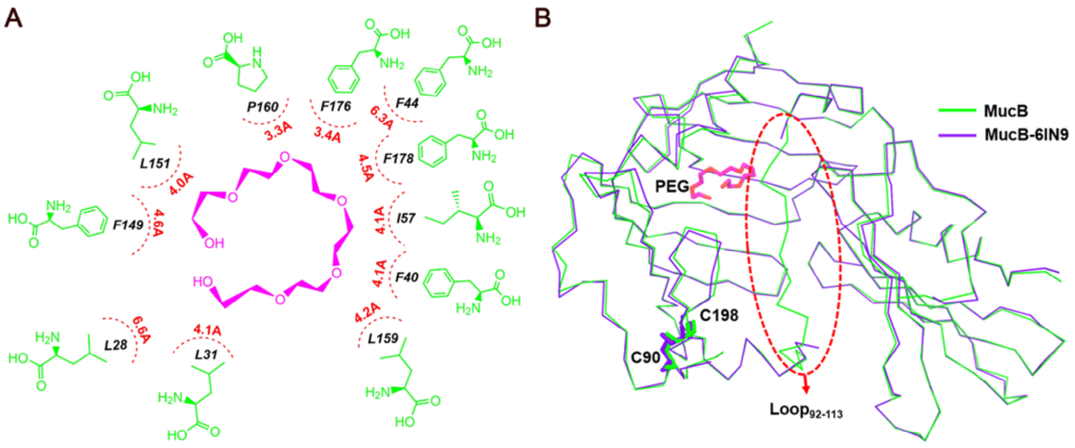

Supplementary Figure 4. (A) A schematic of the hydrophobic cavity in MucB, the polyethylene glycol (PEG) molecule is shown with pink stick and the distances between key residues and PEG molecule are shown. (B) Overview of superimposed of MucB and MucB-6IN9. A bound PEG molecule in our structure is shown as hot-pink sticks. The region of the dashed red is the position of $loop_{92\text{-}113}$. In the structure of MucB-6IN9, $loop_{92\text{-}113}$ is too flexible to be detected.

Supplementary Figure 5.

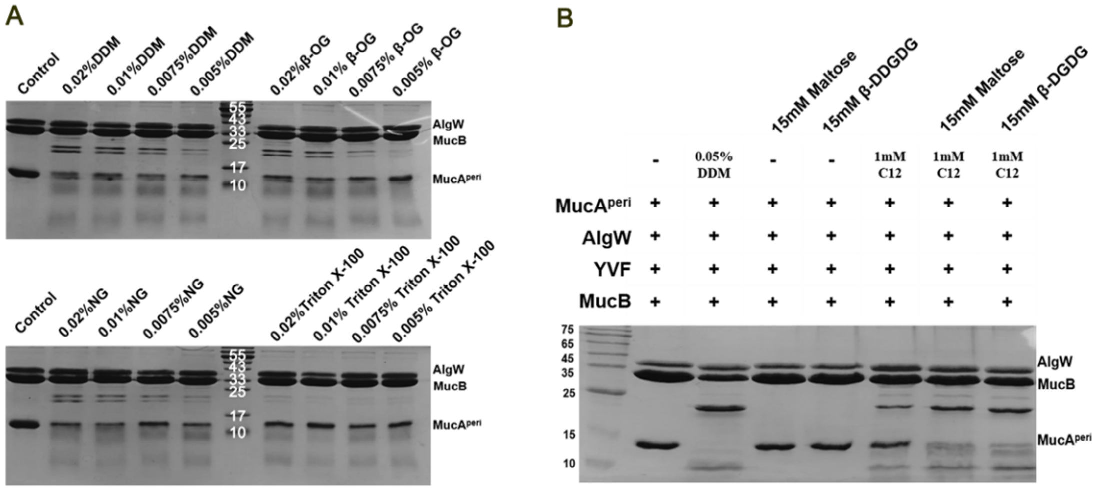

Supplementary Figure 5. (A) SDS-PAGE assay of MucA^{peri} (125 μM) degradation by AlgW (25 μM) in the presences of MucB (130 μM), YVF peptide (80 μM), and different detergent concentration (DDM, NG, β-OG and TritonX-100). The fractions were incubated in degradation buffer (25mM Tris-HCl, PH 7.5, 150 mM NaCl) at 37℃ for 30 min. (B) The lauric acid (C12)-induced MucA^{peri} cleavage process was obviously accelerated by adding disaccharides (15mM Maltose or β-D-Glucopyranosyl-D-glucose(β-DGDG)), the fractions were incubated in degradation buffer (25mM Tris-HCl, PH 7.5, 150 mM NaCl) at 37℃ for 20 min.

Supplementary Figure 6.

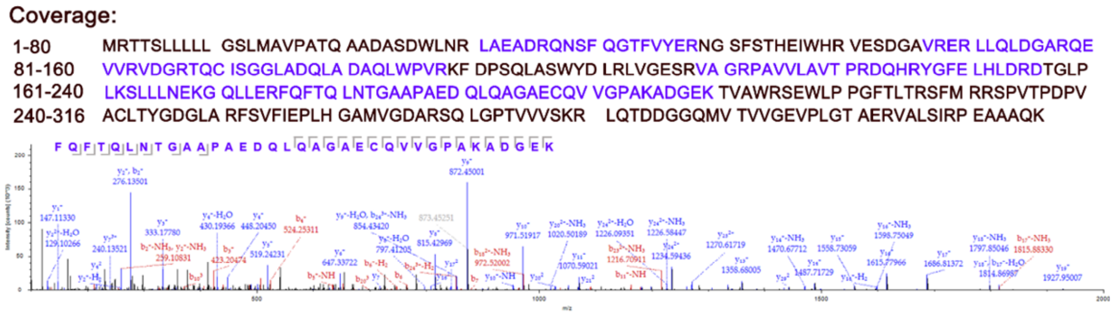

Supplementary Figure 6. Identification of cleavage fragments by Mass Spectrometry. Top: The sequence identified after standard trypsin digestion and mass spectrometry had high sequence coverage with MucB. The detected sequence was colored in blue. Bottom: The Mass spectrogram of a representative detected fragment sequence "QLLERFQFTQLNTGAAPAEDQLQAGAECQVVGPAKADGEK".

Supplementary Figure 7.

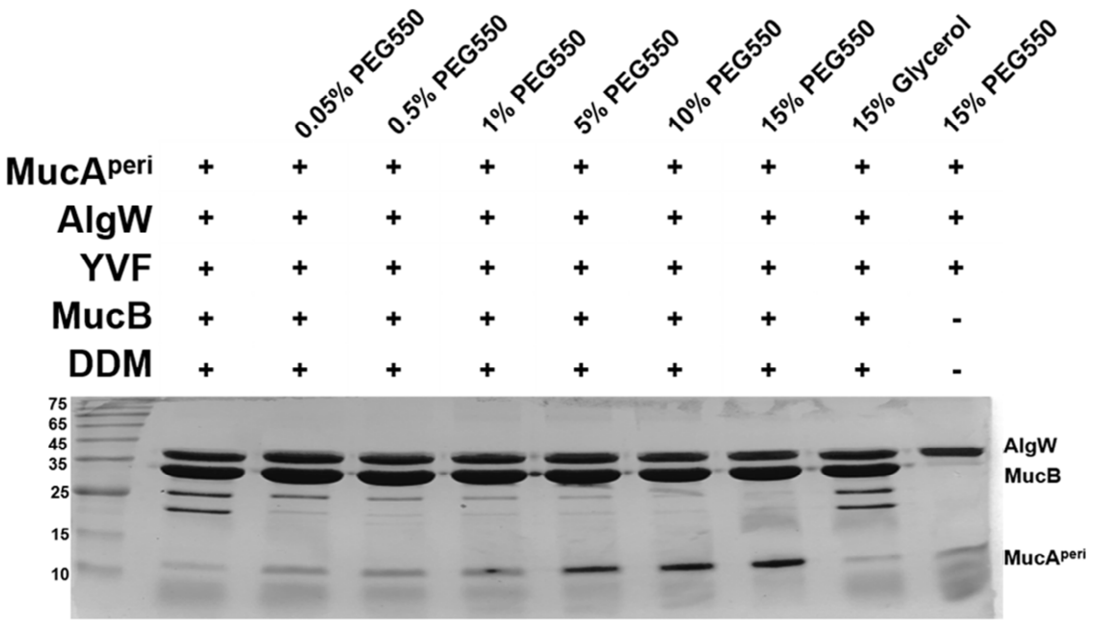

Supplementary Figure 7. PEG competed with the DDM for binding site in hydrophobic cavity of MucB and the protective effect was in a concentration-dependent manner. Gradient concentration of PEG550 (0.05%, 0.5%, 1%, 5%, 10%, 15%) was added in reaction system (125 μM MucA^{peri}, 25 μM AlgW, 130 μM MucB, 80 μM YVF peptide, and 0.05% DDM) and incubated at 37□ for 30 min. As negative control, the penultimate column was performed by adding 15% glycerol in reaction system. The last column reaction system (without the protection of MucB) was positive control.

Supplementary Figure 8.

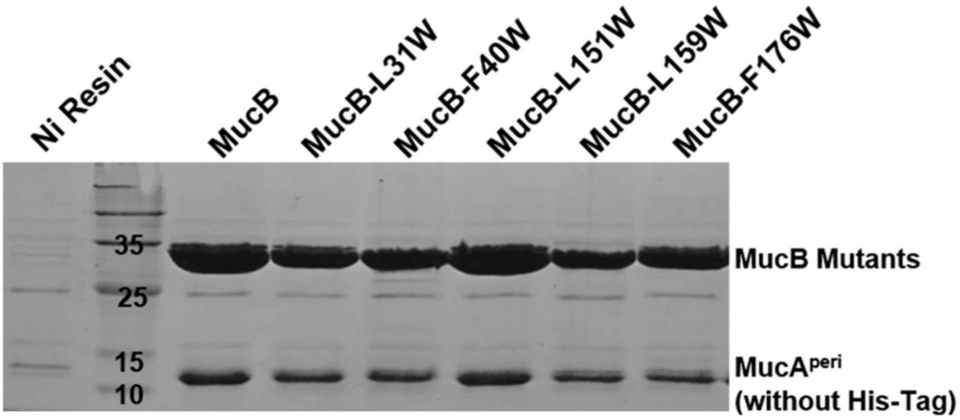

Supplementary Figure 8. His-affinity Pull-down assay. Incubate 75 µg MucB or its variants (with His-tag) with excessive $MucA^{peri}$ (without His-tag, 72 µg), the molar ratio of $MucA^{peri}$ to MucB was 3:1 to ensure excess of $MucA^{peri}$ interact with MucB or mutants. Fractions were eluted with solution buffer containing 300 mM imidazole and determined by 15% SDS-PAGE gel and visualized by Coomassie Brilliant Blue stain.

Supplementary Figure 9.

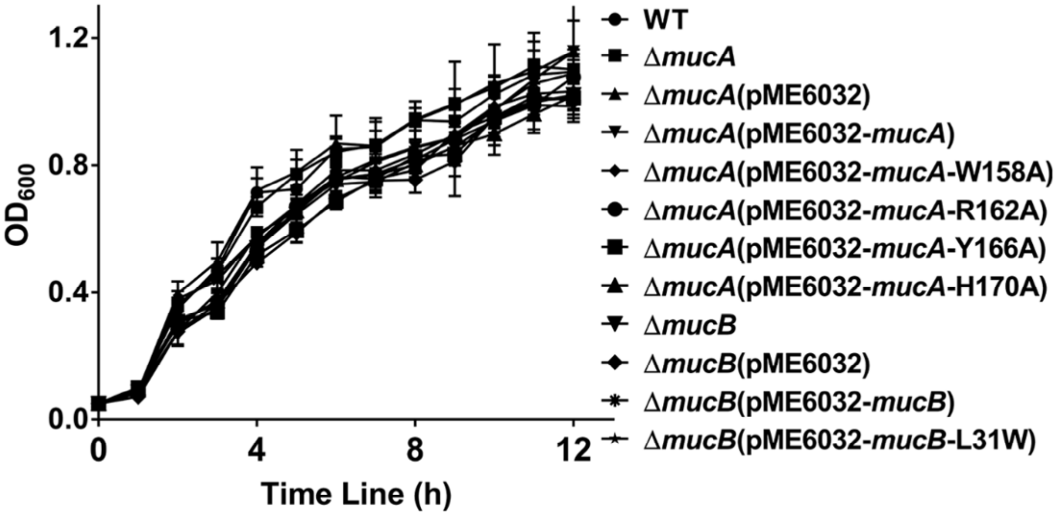

Supplementary Figure 9. The conventional growth curve determination of *P. aeruginosa* PAO1 and all mutations. Mutations exhibited no growth defect relative to *P. aeruginosa* PAO1. Measurements were performed three times.

Supplementary Tables

Supplementary Table 1. Data collection and refinement statistics (molecular replacement)

Data collection and structure refinement statistics of MucA^{peri}-MucB complex.

**Data collection**	
Space group	$C222_1$
**Cell dimensions**	
a, b, c (Å)	71.907 186.429 50.628
α, β, γ (°)	90 90 90
Wavelength (Å)	0.97853
Resolution (Å)[a]	35.95-1.905(1.973-1.905)
R_{sym}	0.141(0.454)
Average I/σ（I）	15.57(4)
R_{meas}	0.145(0.486)
R_{pim}	0.042(0.174)
Completeness (%)	98.90(92.26)
$CC_{1/2}$	0.746
Redundancy	10(7.2)
**Refinement**	
Resolution (Å)	35.95-1.905(1.973-1.905)
No. of reflections	26847（2455）
R_{work}/R_{free}	0.1729/0.1929 (0.2085/0.2096)
**No. of atoms**	
Protein	330
Ligand/ion	26
Water	186
B-factor from Wilson plot (Å^2)	16.93
Average B-factor	25.2
Protein	24.3
Ligand/ion	44.1
Water	34.9
**r.m.s.d.**[b]	
Bond lengths (Å)	0.028
Bond angles (°)	1.92
Number of TLS groups	1
**Ramachandran plot** （favored/allowed/ outliers）	97.84/2.16/0

a Values in parentheses are for the highest resolution shell.
b r.m.s.d. indicates root mean square deviation.

Supplementary Table 2. Bacterial strains, plasmids, constructs, oligos and sequences.

Bacterial strains and plasmids

Strains/Plasmids	Genotype /phenotype	Source/reference
Pseudomonas aeruginosa	strain ATCC 15692 /PAO1	Laboratory collection
Escherichia coli	strain K12	Laboratory collection
S17-1 Chemically Competent Cell（ZC1035 ）	RP4-2(Km::Tn7, Tc::Mu-1), pro-82, LAMpir, recA1, endA1, thiE1, hisdR17, creC5110	Reference 23
pET22b	lac operator, cspA promoter, Amp^r	Laboratory collection
pCold	T7/laco promoter, ColE1 ori， F1 ori, Amp^r	Laboratory collection
pEX18Gm	lac operater, SacB Promoter, Gm^r	Reference 23
pME6032	Tac promoter, pVS1 ori, p15A ori, Tet^r	Reference 24

Constructs, oligos and sequences

Constructs	oligos and sequences (5’ to 3’)	Source/reference
pET22b::MucA^{peri}(106-194)-F	5' TTAAGAAGGAGATATACATATGTACAACCAGAACGACGCCCTGC 3'	This work
pET22b::MucA^{peri}(106-194)-R	5' GGTGGTGGTGGTGGTGCTCGAGGCGGTTTTCCAGGCTGGCTGCC 3'	This work
pET22b::MucB(Full Length)-F	5' TTAAGAAGGAGATATACATATGATGCGCACCACCTCCCTGTTGC 3'	This work
pET22b::MucB(Full Length)-R	5' GGTGGTGGTGGTGGTGCTCGAGTTTCTGGGCGGCGGCCTCGGGC 3'	This work
pEX18Gm::MucA-F	5' AACGACGGCCAGTGCCAAGCTTCCCTGAGCCCGATGCAATCCATT 3'	This work
pEX18Gm::MucA-R	5' TTCGAGCTCGGTACCCGGGGATAACCATGGCACCGTGCAGCGGCT 3'	This work
pEX18Gm::MucB-F	5' AACGACGGCCAGTGCCAAGCTTTCCAGCAGTTGCCCGAGGATTTG 3'	This work
pEX18Gm::MucB-R	5' TTCGAGCTCGGTACCCGGGGATGATGATGAAGCCGGAACCCAGCG 3'	This work
pEX18Gm::ΔMucA-F	5' GGAGAGACATGCGCACCACCTCCCTGTTGCTTTTGCTTGGCAGCC 3'	This work
pEX18Gm::ΔMucA-R	5' AGCGATACCTCTCTTGGCATTTGCCGCTGTGTCAGGCTTCTCGCA 3'	This work
pEX18Gm::ΔMucB-F	5' TCGAGGAGCAGGGGCGAGTGGTGGCGACCGAGCCGGGAGCGGTAT 3'	This work
pEX18Gm::ΔMucB-R	5' GTCTCTCCTCAGCGGTTTTCCAGGCTGGCTGCCCGAGCGTAGGGC 3'	This work
pME6032::MucA-F	5' CCGCTCGAGATGAGTCGTGAAGCCCTGCAGG 3'	This work
pME6032::MucA-R	5' CCGGAATTCTCAGCGGTTTTCCAGGCTGGCT 3'	This work
pME6032::MucB-F	5' CCGCTCGAGATGCGCACCACCTCCCTGTTGC 3'	This work
pME6032::MucB-R	5' CCGGAATTCTCAGTGGTGGTGGTGGTGGTGC 3'	This work
pCold::MucA$_{(106-180)}$-F	5' TCATCATCATCATCATATGGCCGCCATGAACCAGAACGACGCCC 3'	This work
pCold::MucA$_{(106-180)}$-R	5' GCTTTTAAGCAGAGATTACCTACTCTGTACCACTGACGGCGGAT 3'	This work
pET22b::MucA(S153A)-R	5' GGACGAGGAGTTGGTGATCACC 3'	This work
pET22b::MucA(S153A)-F	5' GCCGATACCCGCTGGCATGAGC 3'	This work
pET22b::MucA(W158A)-R	5' GCGGGTATCGCTGGACGAGGAG 3'	This work
pET22b::MucA(W158A)-F	5' GCCCATGAGCAGCGTCTGCCGA 3'	This work
pET22b::MucA(H159A)-R	5' CCAGCGGGTATCGCTGGACGAG 3'	This work
pET22b::MucA(H159A)-F	5' GCCGAGCAGCGTCTGCCGATCT 3'	This work
pET22b::MucA(R162A)-R	5' CTGCTCATGCCAGCGGGTATCG 3'	This work
pET22b::MucA(R162A)-F	5' GCCCTGCCGATCTACCTGCGTC 3'	This work
pET22b::MucA(Y166A)-R	5' GATCGGCAGACGCTGCTCATGC 3'	This work
pET22b::MucA(Y166A)-F	5' GCCCTGCGTCAGCACGTGCAAC 3'	This work
pET22b::MucA(R168A)-R	5' CAGGTAGATCGGCAGACGCTGC 3'	This work
pET22b::MucA(R168A)-F	5' GCCCAGCACGTGCAACAATCCG 3'	This work
pET22b::MucA(Q169A)-R	5' ACGCAGGTAGATCGGCAGACGC 3'	This work
pET22b::MucA(Q169A)-F	5' GCCCACGTGCAACAATCCGCCG 3'	This work
pET22b::MucA(H170A)-R	5' CTGACGCAGGTAGATCGGCAGACGCTGCTC 3'	This work
pET22b::MucA(H170A)-F	5' GCCGTGCAACAATCCGCCGTCAGTGGTACA 3'	This work
pET22b::MucA(Q172A)-R	5' CACGTGCTGACGCAGGTAGATC 3'	This work
pET22b::MucA(Q172A)-F	5' GCCCAATCCGCCGTCAGTGGTA 3'	This work
pET22b::MucA(Q173A)-R	5' TTGCACGTGCTGACGCAGGTAG 3'	This work
pET22b::MucA(Q173A)-F	5' GCCTCCGCCGTCAGTGGTACAG 3'	This work
pET22b::MucA(S177A)-R	5' GACGGCGGATTGTTGCACGTGC 3'	This work
pET22b::MucA(S177A)-F	5' GCCGGTACAGAGAGCGCGCTGC 3'	This work
pET22b::MucA(E180A)-R	5' TGTACCACTGACGGCGGATTGTTGCACGTG 3'	This work
pET22b::MucA(E180A)-F	5' GCCAGCGCGCTGCCCTACGCTCGGGCAGCC 3'	This work
pET22b::MucA(Y185A)-R	5' GGGCAGCGCGCTCTCTGTACCACTGACGGC 3'	This work
pET22b::MucA(Y185A)-F	5' GCCGCTCGGGCAGCCAGCCTGGAAAACCGC 3'	This work
pET22b::MucA(R187A)-R	5' AGCGTAGGGCAGCGCGCTCTCT 3'	This work
pET22b::MucA(R187A)-F	5' GCCGCAGCCAGCCTGGAAAACC 3'	This work
pET22b::MucA(S190A)-R	5' GGCTGCCCGAGCGTAGGGCAGC 3'	This work
pET22b::MucA(S190A)-F	5' GCCCTGGAAAACCGCCTCGAGC 3'	This work
pET22b::MucB(L31W)-F	5' TGGGCCGAGGCCGATCGCCAGA 3'	This work
pET22b::MucB(L31W)-R	5' ACGATTCAGCCAGTCGGAAGCG 3'	This work
pET22b::MucB(F40W)-F	5' TGGCAAGGCACCTTCGTCTACG 3'	This work
pET22b::MucB(F40W)-R	5' ACTGTTCTGGCGATCGGCCTCG 3'	This work
pET22b::MucB(L151W)-F	5' TGGCACCTGGACCGCGACACCG 3'	This work
pET22b::MucB(L151W)-R	5' CTCGAAGCCGTAGCGATGCTGG 3'	This work
pET22b::MucB(L159W)-F	5' TGGCCGTTGAAGTCGCTGCTGC 3'	This work
pET22b::MucB(L159W)-R	5' GCCGGTGTCGCGGTCCAGGTGC 3'	This work
pET22b::MucB(F176W)-F	5' TGGCAGTTCACCCAGTTGAATA 3'	This work
pET22b::MucB(F176W)-R	5' GCGCTCGAGCAACTGCCCCTTC 3'	This work
pET22b::MucB-NTD-F	5' CTCGAGCACCACCACCACCACC 3'	This work
pET22b::MucB-NTD-R	5' GACCTGGCATTCGGCGCCCGCC 3'	This work
pET22b::MucB-NTD-P106A-F	5' CCACAGCTGGGCATCGGCCAGTTGG 3'	This work
pET22b::MucB-NTD-P106A-R	5' GCCGTGCGCAAGTTCGATCCCTCCC 3'	This work
pET22b::MucB-NTD-P112A-F	5' ATCGAACTTGCGCACCGGCCACAGC 3'	This work
pET22b::MucB-NTD-P112A-R	5' TACTCCCAGCTGGCTTCCTGGTACG 3'	This work

Methods

Sample preparation, Trypsin digestion and Mass spectrometry analysis

The mixture of MucB, AlgW, YVF peptide and 0.05%DDM were incubated in a lysis buffer consisting of 25 mM Tris–HCl (pH 7.5), 150 mM NaCl, 5 % glycerol at 37□ for 1h. Then in-gel digestion was performed using a method described [1,2]. Briefly, the lower band (around 25 KDa) was cut from the gels, and the gel pieces were washed three times for 15 min each with 200 μl of 50 mM ammonium bicarbonate with 50% (v/v) acetonitrile and then dried under vacuum. Then, the samples were reduced with 10 mM TCEP at 56°C for 1 h, and alkylated with 40mM CAA in the dark for 45 min. After reduced and alkylated, the colloidal particles were eluted with 25mM NH_4HCO_3 for three times, and dehydrated with 50% ACN, 100% CAN until completely dried. Trypsin (1:50) was added to digest the proteins at 37°C overnight. Peptides were extracted with 5 μl of extracting solution (50% (v/v) acetonitrile and 0.3% (v/v) trifluoroacetic acid) for 10 min by sonication. Finally, the mass spectra were obtained using a liquid chromatography mass spectrometer (LC /MS) analyzer (orbitrap fusion lumos, Thermo Scientific), and searched the protein database of *Pseudomonas aeruginosa* PAO1 with the search through NCBI databases.

References

1 Matsuura, K., Kadoyama, K. & Matsuyama, S. The influence of chronic nicotine treatment on proteins expressed in the mouse hippocampus and cortex. *European journal of pharmacology* **780**, 16-25, doi:10.1016/j.ejphar.2016.03.025 (2016).

2 Reginald, K. & Chew, F. T. The major allergen Derp2 is a cholesterol binding protein. *Sci Rep* **9**, 1556, doi:10.1038/s41598-018-38313-9 (2019).
